# Assessing Willingness to Pay for IVF Among Infertile Women in Greece: A Single‐Center Case Study

**DOI:** 10.1002/hsr2.70402

**Published:** 2025-01-26

**Authors:** Christos Ntais, Mary Artsita, Michael A. Talias, John Fanourgiakis, Nikolaos Kontodimopoulos

**Affiliations:** ^1^ Epidemiology Program, School of Science and Technology Hellenic Open University Patras Greece; ^2^ Healthcare Management Program, School of Economics & Management Open University of Cyprus Nicosia Cyprus; ^3^ Healthcare Management Program, School of Social Sciences Hellenic Open University Patras Greece; ^4^ Department of Management Science and Technology Hellenic Mediterranean University Agios Nikolaos Crete Greece; ^5^ Department of Economics and Sustainable Development Harokopio University Athens Greece

**Keywords:** case study, contingent valuation, in vitro fertilization, infertility, willingness to pay

## Abstract

**Background and Aims:**

In recent years, In Vitro fertilization (IVF) science has grown by leaps and bounds in the field of assisted reproduction, helping millions of couples worldwide. The aim of this study is to examine the extent to which infertile women are willing to pay for IVF services in Greece.

**Methods:**

Through the distribution of questionnaires, willingness to pay (WTP) is recorded according to IVF success rates, and the relationship between WTP and the respondents' demographic characteristics is analyzed. Subjects were divided into two groups according to age and were given a hypothetical scenario, according to the contingent valuation approach.

**Results:**

The majority of women were reluctant to pay the amount of money corresponding to the average cost of an IVF cycle in Greece. In particular, participants replied that the amount they would be willing to pay is only one‐third of the indicative amount of €6000. Moreover, WTP is not influenced by the professional status or educational level of the participating women.

**Conclusion:**

This study highlights the financial barriers many couples face when pursuing assisted reproduction. If the average cost of IVF were lower, infertile women would be willing to undergo more IVF cycles if necessary. This finding has important implications for the valuation of IVF services and the development of pricing policies to enhance affordability and accessibility. Policymakers must consider reforms that balance the cost of IVF with its societal benefits. By incorporating this aspect into pricing and policy decisions, Greece can improve the accessibility of IVF services and ensure equitable treatment opportunities for all concerned individuals.

## 1 Introduction

Since 1950, there has been a global downward trend in births. On average, the worldwide ratio has declined from 4.86 births per woman in 1950 to 2.32 in 2021 [1]. Although infant mortality is declining, the global downward trend in births is driven by fewer births per woman [2]. In addition, there has been an increase in infertility globally, rendering the acquisition of children a challenge for modern couples [3]. In a recent survey, the World Health Organization reports that one in six people, or about 17.5% of the population, will face infertility at some point in their lives [4]. When assessing a number of studies on the prevalence of infertility, Boivin et al. [5] reported that, in more developed countries, 12‐month infertility rates ranged from 3.5% to 16.7%. Additionally, they found that while 56.1% of infertile people or couples sought medical care on average, only 22.4% of them were actually treated. According to Vander Borght and Wyns [6] and Inhorn and Patrizio [7], the prevalence of infertility in couples of reproductive age varies between 8% and 12% worldwide. Between 1990 and 2017, there was a global increase in infertility by 14.96% for women and 8.22% for men [8]. The inability or difficulty of having a child can cause psychological, social, and economic problems for the couple. According to a study conducted in Denmark, couples experiencing infertility problems that prevent them from having children are three times more likely to divorce than those who have had a child [9].

The funding of infertility treatments varies globally; some countries fully or partially cover the costs under certain conditions, while others require individuals to bear the expenses themselves. Willingness to pay (WTP) estimates can be a robust means of assessing the value payers attach to assisted reproductive technology (ART) procedures such as In Vitro fertilization (IVF), where societal decisions are made about funding infertility treatments [10]. WTP refers to the maximum amount someone is willing to pay for a product or service to achieve a specific desired outcome. This can be a patient, a health system (such as national or insurance‐based funding schemes), or a mix of the two [11]. WTP per quality‐adjusted life year (QALY) estimations have been developed in a number of countries to ascertain the amount of money that the general public or individual patients are willing to pay for medical treatments. There is little consensus about the best way to calculate these thresholds, whether or not to make them explicit, and how to use them in real‐world scenarios, despite the fact that they could be helpful when assessing “value‐for‐money” [12]. However, when deciding on health policy, a number of countries and international organizations—including the World Health Organization—have implemented these thresholds [13, 14].

In 1994, just 15 years after the birth of the first child through IVF, a study was conducted in Massachusetts, USA, on the WTP for the procedure, which showed that the average WTP for IVF from 150 couples surveyed was $17,730 for a success rate of only 10% [15]. They were also willing to pay an annual tax of $32 to make the assisted reproductive procedure a public good for 1200 Massachusetts couples annually. A year later, a similar survey was carried out in Sweden which lasted 4 months and asked infertile couples to rate the benefit of assisted reproduction through WTP [16]. The results showed that most couples who participated in the survey stated that they were willing to pay more than the average cost for the IVF procedure, including labor.

A recent time trend analysis of births in Greece indicated a dramatic decrease in natality, predominantly attributed to the large decline in births in the 1980s, which could not be reversed in the 1990s and 2000s [17]. The recent decrease in births was associated with the financial recession and has put the Greek population in a detrimental low‐fertility spiral. The WTP for IVF services is a crucial area of research, particularly in Greece, for several reasons. First, Greece has one of the lowest fertility rates in Europe, making assisted reproduction an essential healthcare service for many couples [18]. IVF services are primarily offered through a mix of public and private healthcare providers. Public healthcare facilities often have long waiting times and limited resources, prompting many couples to seek treatment in private infertility centers, where the costs are significantly higher [19]. The Greek health system provides partial insurance coverage for IVF, typically covering certain medications but not the full cost of treatment cycles. This leaves patients responsible for substantial out‐of‐pocket expenses, which can be a major barrier to access.

The aim of this stated‐preference study was to examine WTP for IVF and its associated factors, among infertile women up to 40 years old in Greece according to the contingent valuation approach. By providing empirical data on the affordability of IVF services and identifying potential gaps between the demand for and the accessibility of such services, this study aspires to inform policymakers and healthcare providers about the necessity of cost‐reduction strategies or subsidization programs to improve access to IVF.

## 2 Methods

### 2.1 Study Design

The study employed the contingent valuation method, a well‐established approach for assessing individuals' WTP for nonmarket goods and services, such as healthcare. This method was chosen for several reasons:

i. Suitability for hypothetical scenarios. The contingent valuation method allows researchers to present hypothetical scenarios to participants, simulating real‐world decision‐making processes regarding their WTP for IVF services. This approach is particularly useful in healthcare settings where direct price experiments may be impractical or unethical.
ii. Flexibility in capturing preferences. The contingent valuation method provides flexibility in tailoring scenarios to include different success rates and cost levels of IVF, which are critical factors influencing WTP. This customization ensures that valuation reflects realistic considerations and aligns with participants' personal circumstances.
iii. Widespread use in health economics. The contingent valuation method is widely recognized and frequently used in health economics to evaluate WTP for medical treatments and public health interventions. Its extensive application and validation in similar contexts enhance the reliability and comparability of the present study's findings.

### 2.2 Sample and Data

A convenience sample of 201 women aged 18–40 years old who live in Greece and speak Greek was recruited for the present study which was conducted between January and March 2023. These women were infertile and were in the process of IVF with their own eggs at a private fertility clinic based in Athens, Greece. Male partners were excluded from the study, as the majority were reluctant to participate. One of the main factors affecting the success of IVF is age [20, 21]. The older a woman is, the less likely she is to succeed with assisted reproduction. For this reason, it was considered important to separate the sample into two age groups in order to tend the questions as closely as possible to the actual situation of the respondents. Older women were excluded from the survey as the chances of success of an IVF cycle were considerably lower. Also, a large proportion of older women who wish to have a child will need either a sperm donor, an egg donor, or a surrogate mother. These procedures differ from IVF alone, and their costs are much higher. As a result of the foregoing, it was impossible to create scenarios for each individual case.

The process of collecting the sample and, by extension, the data were done in two ways. First, the questionnaire was converted into an electronic format using Google Forms. It was then sent for completion to infertile women who visited the said clinic. The questionnaire was also printed and distributed to patients at the same clinic, with the assistance of gynecologists and IVF practitioners. Through the combination of the two aforementioned sampling methods, the aim of achieving greater diversity in the final sample was achieved. Finally, it should be noted that the women who were invited to respond were informed about the purpose of the survey, the protection of their personal data and their voluntary participation. During the hypothetical scenario for the WTP study, participants were informed that their answers would not affect the care services they would receive.

### 2.3 Research Instrument

**Figure 1:**
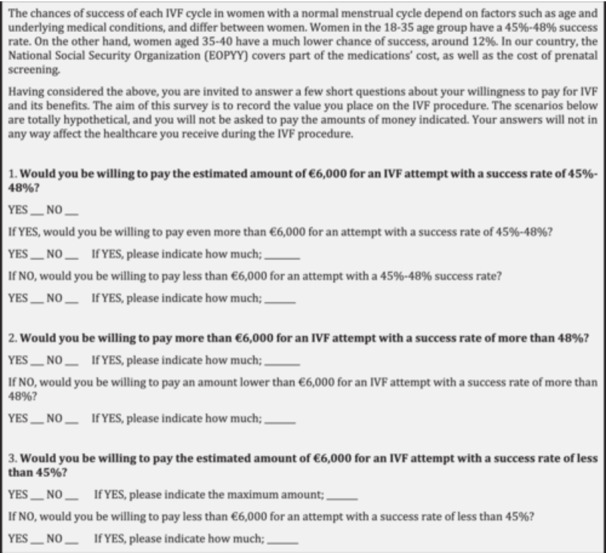
The willingness to pay questions for the 18–35 age group.

The women were divided into two age groups, 18–35 and 36–40, upon consultation with the IVF experts of the subject clinic, who suggested an experiential success rate of 45%–48% for the 18–35 group and 12% for the 36–40 group. The questionnaire, which was designed and used for the purposes of this study, consisted of two groups of items. The first group included demographic, social, and psychometric questions. More specifically, the respondents' demographics were recorded, including age, place of origin, professional status, annual personal income, family status, and educational level. Then, a number of scale questions were used to examine their different life aspects and the feelings they experienced during the IVF procedure. The scale used for the psychometric questions, such as support from partners and family/friends, was from 1 to 10, with 1 being the *minimum* and 10 being the *maximum*. Other closed‐type questions involved the IVF procedure, such as how many times they had undergone it and how many times they would be willing to undergo this procedure. The second group of questions were related to WTP. The WTP questions for the 18–35 age group are presented in Figure 1.

The questions for the 36–40 age group were the same, but with a success rate of 12% instead of 45%–48%. There is currently a lack of clear WTP thresholds expressing how much money payers are maximally willing to pay for an infertility treatment that results in pregnancy or live birth [22], and there are variations in the threshold methodologies used across various studies [10]. In this study, an estimated WTP threshold of €6000 was used which is compatible with previous research in this field [23, 24].

To enhance the content validity and reliability of the questionnaire, several quality assurance measures were implemented:

i. Pretesting the questionnaire: Before the main survey, the questionnaire was pretested on a small sample of 20 participants who resembled the target population in terms of demographics and IVF‐related experiences. Pretesting was conducted to ensure that all questions were clear, understandable, and relevant to the study objectives. Feedback from the pretest group led to several refinements, including simplifying complex terms, adjusting the wording of hypothetical scenarios, and ensuring that response options were comprehensive and unambiguous.
ii. Expert review: The questionnaire was reviewed by a panel of experts in health economics, fertility treatments, and survey design. Their input helped to align the questions with the contingent valuation method's principles and ensured that key aspects, such as IVF success rates and costs, were accurately represented.
iii. Pilot study: Following the pretest, a pilot study involving 50 participants was conducted to validate the refined questionnaire. This step confirmed that respondents understood the hypothetical scenarios and were comfortable providing their WTP estimates.

These measures ensured that the questionnaire was both credible and comprehensible for respondents, enhancing the quality and validity of the data collected in this study.

### 2.4 Code of Ethics

The study was approved by the competent body of the Postgraduate Program of Studies “Healthcare Management” of the Hellenic Open University (Institutional Review Board approval reference number: 148349/3‐10‐2022). The research was conducted in accordance with the Declaration of Helsinki. The written informed consent of all participants was secured prior to their inclusion in the study.

### 2.5 Data Analysis

Absolute and relative frequencies were used to describe the qualitative (categorical) variables, while mean values and standard deviations were used for measurements of quantitative (continuous) variables. *T*‐tests were conducted for two independent samples to differentiate views according to age group. In addition, the effect of demographic characteristics on WTP was examined using the Pearson chi‐square independence test. Analysis of variance was applied to differentiate monetary values according to demographic characteristics, followed by multiple comparison tests using Bonferroni's criterion. Finally, correlation tests were performed using Spearman's Rho correlation coefficient. The significance level was set at 0.05, and all statistical tests were two‐sided. The analysis was performed using the SPSS v26.0 software.

## 3 Results

### 3.1 Descriptive Statistics

Out of 225 women approached, 201 agreed to participate in the study (response rate 89.3%). The mean age of the sample was 35.8 ± 3.3 years (range 18–40 years). Regarding residence, 40.8% of the women were living in the greater Athens area. Educational attainment was high as 67.2% of the sample had attended tertiary education. Almost half of the women in the sample were private sector employees (44.3%) with an annual net income between €10,000 and €20,000 (50.2%). The vast majority (74.6%) were married and without children. The sociodemographic characteristics of the sample are presented in Table 1.

**Table 1:** Demographics of the sample (*N* = 201).

Demographics		*N*	%
Age	18–35 years	85	42.3
	36–40 years	116	57.7
Education level	Primary education	4	2.0
	Secondary education	62	30.8
	Tertiary education	83	41.3
	Master's degree	36	17.9
	Doctorate	16	8.0
Work status	Farmer	3	1.5
	Unemployed	14	7.0
	Public servant	34	16.9
	Freelancer	28	13.9
	Private employee	89	44.3
	Domestic activities	33	16.4
Personal annual income	€0–€10,000	65	32.3
	€10,000–€20,000	101	50.2
	€20,000–€30,000	32	15.9
	€30,000–€40,000	3	1.5
Family status	Single w/children	3	1.5
	Single w/o children	20	10.0
	Married w/o children	150	74.6
	Married w/children	28	13.9

### 3.2 Willingness to Pay

#### 3.2.1 Age Group 18–35

This age subgroup consisted of 85 women, 59 of whom stated that they would be willing to pay the estimated amount of €6000 for an IVF attempt with a success rate of 45%–48%, while 22 of those 59 would be willing to pay an even higher amount of money for an attempt with the same success rate. In this group, most of the subjects stated their WTP up to €10,000 (mean = €8590), while only two women were willing to pay even more money. Approximately half of the women unwilling to pay €6000, reported that they would pay less than €6000 for an IVF attempt with a success rate of 45%–48%. Most of these women would pay less than €3000 (mean = €2107). When asked about their WTP, which was even more than €6000 for a chance of success higher than 48%, 62 women answered positively. Apparently, most would pay €7000 (mean = €8237), while only 3 women would pay much more money (€15,000). Almost all the women in the 18–35 age group would pay less than €6000 for an IVF attempt with a chance of success higher than 48%. Most of these women provided responses between €2000 and €3000, with an average amount of €2336. When asked if they would be willing to pay the estimated amount of €6000 for an IVF attempt with a less than 45% chance of success, only 16 women responded positively. Furthermore, these women answered that they would be willing to pay up to an average of around €6600. Only seven more positive responses occurred when the probability of success remained the same (< 45%), while the amount of payment below €6000 was reduced. This group of women responded that they would be willing to pay an average of €2004. Table 2 summarizes the responses of women aged 18–35 years.

**Table 2:** WTP for IVF—Age Group 18–35 years (*Ν* = 85).

	Scenario	Participants		Mean amount (€)
		*N*	%	
1	Willing to pay €6000 for 45%–48% success rate	59	69.4	6000
2	Willing to pay > €6000 for 45%–48% success rate	22	25.9	8590
3	Willing to pay < €6000 for 45%–48% success rate	14	16.5	2107
4	Willing to pay > €6000 for > 48% success rate	62	72.9	8237
5	Willing to pay < €6000 for > 48% success rate	83	97.6	2336
6	Willing to pay €6000 for < 45% success rate	16	18.8	6600
7	Willing to pay < €6000 for < 45% success rate	23	27.1	2004

#### 3.2.2 Age Group 36–40

This age subgroup included 116 women. Among them, 50 indicated WTP an estimated amount of €6000 for an IVF attempt with a 12% success rate. Additionally, 24 expressed WTP an even higher amount for the same success rate, with most of this group willing to spend up to €10,000 (mean = €8495). Of the 66 women unwilling to pay €6000, 29 were open to paying less than €6000 for an IVF attempt with a 12% success rate, typically within the range of €2000–€3000 (mean = €1815). Regarding WTP over €6000 for a success rate higher than 12%, 79 women responded affirmatively. Most of these women would pay €7000–€8000 (mean = €7981), while 12 would spend up to €10,000. For the 37 women reluctant to pay over €6000 for a success rate higher than 12%, 25 were willing to pay less, with an average amount of €2200. Most in this group cited a range of €1000–€3000. When asked about paying €6000 for an IVF attempt with a success rate below 12%, only 18 women responded positively, with an average WTP of €7166. Among 98 women who replied negatively to the previous question, 28 were willing to pay an average of €1826 for the same probability of success (< 12%). Table 3 summarizes the responses of women aged 36–40 years.

**Table 3:** WTP for IVF—Age Group 36–40 years (*Ν* = 116).

	Scenario	Participants		Mean amount (€)
		*N*	%	
1	Willing to pay €6000 for 12% success rate	50	43.1	6000
2	Willing to pay > €6000 for 12% success rate	24	20.7	8495
3	Willing to pay < €6000 for 12% success rate	29	25.0	1815
4	Willing to pay > €6000 for > 12% success rate	79	68.1	7981
5	Willing to pay < €6000 for > 12% success rate	25	21.6	2200
6	Willing to pay €6000 for < 12% success rate	18	15.5	7166
7	Willing to pay < €6000 for < 12% success rate	28	24.1	1826

### 3.3 Does Support From a Partner and Family/Friends Affect the Number of IVF Cycles a Woman Is Willing to Undergo?

The analysis of variance, examining differences in the number of IVF cycles that women aged 18–35 were willing to have, depending on their partner's or family/friends' support, did not show statistically significant differences (*F* = 0.145, *p* = 0.93 for partners, *F* = 0.402, *p* = 0.75 for family/friends). The same analysis for the 36–40 age group also showed no statistically significant differences for family support (*F* = 0.927, *p* = 0.43), but a statistically significant difference was identified for the partner's support (*p* < 0.001). Specifically, women who would undergo only two IVF cycles reported lower levels of support (score = 6.87) compared to those who would attempt three (8.37), four (8.92), and five plus (9.26) IVF cycles.

### 3.4 Does Income Affect WTP?

In the 18–35 age group, WTP was independent of income for all, but one, combinations of IVF success rate and amount of money. The only statistically significant association was observed between income and WTP €6000 for a probability of success higher than 48% (Pearson chi‐square, *p* = 0.02). In particular, all the women with an annual personal income of €20,000–€30,000, as well as 78.6% of women with an income of €10,000–€20,000, and 57.6% of women with an income lower than €10,000 stated their WTP this amount of money for a success rate over 48%. Regarding the 36–40 age group, again only one significant association was recorded, as 84% of the women with an annual income of €10,000–€20,000 refused to pay even less than €6000 for a probability of success less than 12% (Pearson chi‐square, *p* = 0.02).

### 3.5 Does Educational Level Affect WTP?

For both age groups under investigation, the Pearson chi‐square tests showed independence between educational attainment and WTP for IVF, for all the combinations of success rate and amount of money.

### 3.6 Does Professional Status Affect WTP?

For both age groups under investigation, Spearman's Rho correlation coefficient showed no significant association between professional status and WTP for IVF, for all the combinations of success rate and amount of money.

## 4 Discussion

The majority of the women participating in our study were reluctant to pay the amount of money corresponding to the average cost of an IVF cycle in Greece. Notably, WTP was related to the success rate of the IVF procedure. A study conducted in Iran in 2019 also confirmed this finding [25]. It should be noted that there was no significant correlation between individual annual income and WTP. The only exception was observed in the 18–35 age group, where income seemed to influence WTP for an IVF cycle with a probability of success higher than 48%. A study conducted in Hong Kong, demonstrated that income was a significant independent determinant of the maximum WTP for IVF and less than half of women with lower income were willing to pay higher [26].

A finding with potentially important implications is that education level does not appear to affect women's WTP for IVF, that is, higher educational attainment was not associated with higher WTP for the procedure. A possible explanation might be that women are not adequately informed about infertility and ART procedures, and this may perhaps eliminate the effect of education on WTP [27]. Contrastingly, a recent study from Brazil showed that the higher the woman's educational level, the higher the WTP for an IVF treatment [28]. In this study, professional status does not seem to affect WTP either, as women with more stable and growing careers were not more willing to pay higher amounts of money compared to women in uncertain career paths.

In the results related to the psychology of the surveyed women, there was a difference in the two age groups in relation to support from partners. More specifically, the partners of women in the 18–35 age group seemed to be more supportive of their wives. In contrast, partners of women in the 36–40 age group offer less support which seems to reinforce women's desire to stop the procedure after a total of two assisted reproduction attempts. This may be due to the fact that men's age affects their sperm quality and, by extension, their fertility. Therefore, older couples are more likely to both have an infertility problem, so mutual support becomes an issue. Furthermore, it has been observed that men know less about IVF and often tend to be much more optimistic than women about the outcome of the procedure [29]. Perhaps this attitude is perceived by older women as less than desirable support.

This study highlights the financial barriers many couples face when pursuing assisted reproduction. Similarly, Sfakianoudis et al. [30] highlighted that the economic recession in Greece led to a decline in the demand for ART, with financial concerns causing couples to postpone or abandon fertility treatments. The results emphasize the need for healthcare providers to recognize financial constraints as a key determinant of treatment accessibility. Policymakers must consider reforms that balance the cost of IVF with its societal benefits, including measures for declining birth rates and supporting reproductive rights. By incorporating the findings of this study into pricing and policy decisions, Greece can improve the accessibility of IVF services and ensure equitable treatment opportunities for all concerned individuals. For instance, government‐supported subsidies or insurance coverage for IVF could alleviate the financial burden on individuals and make treatment accessible to a broader population. Furthermore, healthcare providers could adopt tiered pricing structures, where costs are adjusted based on the household income and the number of cycles undertaken. This approach could encourage more couples to pursue additional cycles without the fear of financial exhaustion.

This research is subject to some limitations. In general, stated‐preference studies and specifically contingent valuations are associated with various limitations, such as decisions not having monetary consequences since decisions are not incentivized in contrast with revealed‐preference methods [31]. This is known to lead to hypothetical bias. In addition, there is a general consensus that discrete choice experiments, which are also a stated‐preference approach, are superior to contingent valuation studies. This could be a potential reason for the lack of association of WTP with income in the present study.

The sample of this study consisted of infertile women. Other previous similar studies have investigated the WTP of infertile couples. In the present research, it was not possible in most of the cases to make personal contact with men. From the initial stages, it appeared that women were much more willing to answer the questionnaire compared to the limited number of men who showed willingness to participate. Hence, the male partners were excluded. Another limitation concerned income, in the sense that the related question could have been about family income rather than personal income. It should be noted that IVF success rates are not affected by age only. Other factors associated with IVF success are ovarian reserve or duration of infertility and other patient‐ and cycle‐specific factors [32, 33]. The present study is based on success rates related solely to age. Therefore, there may be women in the research sample with a different success rate than that indicated by their age group. Finally, this study has not investigated the economic impact of different diagnostic methods, such as hysteroscopy, which could be preparatory for IVF, although this aspect has been identified as crucial in determining ART accessibility [34, 35].

## 5 Conclusion

The present study clearly shows that the personal annual income of infertile women in Greece does not affect their WTP for IVF, nor does their professional status, or their educational level either. However, the probability of IVF success is positively correlated with their WTP. In addition, almost all women in both age groups were willing to pay less for an IVF cycle, than the amount initially asked. The money they were willing to pay was about one‐third of the total cost of the IVF procedure. The findings of this study have important implications for the valuation of IVF services and the development of pricing policies to enhance affordability and accessibility. In conclusion, if the cost of IVF in Greece was sufficiently lower, then infertile women would perhaps be willing to try more cycles of the procedure. It is important that assisted reproduction be made accessible to more infertile couples, and IVF not be considered a luxury treatment when infertility is rapidly increasing at the same time.

## Author Contributions

**Christos Ntais:** data curation, formal analysis, writing–original draft. **Mary Artsita:** conceptualization, data curation, formal analysis. **Michael A. Talias:** writing–review and editing. **John Fanourgiakis:** writing–review and editing. **Nikolaos Kontodimopoulos:** conceptualization, writing–review and editing.

## Ethics Statement

All participating women provided their informed consent.

## Conflicts of Interest

The authors declare no conflicts of interest.

## Transparency Statement

The lead author Michael A. Talias affirms that this manuscript is an honest, accurate, and transparent account of the study being reported; that no important aspects of the study have been omitted; and that any discrepancies from the study as planned (and, if relevant, registered) have been explained.

## Data Availability

The data that support the findings of this study are available from the corresponding author upon reasonable request.
